# Orbiting and *In-Situ* Lidars for Earth and Planetary Applications

**DOI:** 10.1109/IGARSS39084.2020.9323088

**Published:** 2021-02-17

**Authors:** Anthony W. Yu, Elisavet Troupaki, Steven X. Li, D. Barry Coyle, Paul Stysley, Kenji Numata, Molly E. Fahey, Mark A. Stephen, Jeffrey R. Chen, Guangning Yang, Frankie Micalizzi, Scott A. Merritt, Robert Lafon, Stewart Wu, Aaron Yevick, Hua Jiao, Demetrios Poulios, Matthew Mullin, Ying Xin Bai, Jane Lee, Oleg Konoplev, Aleksey Vasilyev

**Affiliations:** Lasers & Electro-Optics Branch, NASA Goddard Space Flight Center, Greenbelt, MD 20771 USA; Lasers & Electro-Optics Branch, NASA Goddard Space Flight Center, Greenbelt, MD 20771 USA; Lasers & Electro-Optics Branch, NASA Goddard Space Flight Center, Greenbelt, MD 20771 USA; Lasers & Electro-Optics Branch, NASA Goddard Space Flight Center, Greenbelt, MD 20771 USA; Lasers & Electro-Optics Branch, NASA Goddard Space Flight Center, Greenbelt, MD 20771 USA; Lasers & Electro-Optics Branch, NASA Goddard Space Flight Center, Greenbelt, MD 20771 USA; Lasers & Electro-Optics Branch, NASA Goddard Space Flight Center, Greenbelt, MD 20771 USA; Lasers & Electro-Optics Branch, NASA Goddard Space Flight Center, Greenbelt, MD 20771 USA; Lasers & Electro-Optics Branch, NASA Goddard Space Flight Center, Greenbelt, MD 20771 USA; Lasers & Electro-Optics Branch, NASA Goddard Space Flight Center, Greenbelt, MD 20771 USA; Lasers & Electro-Optics Branch, NASA Goddard Space Flight Center, Greenbelt, MD 20771 USA; Lasers & Electro-Optics Branch, NASA Goddard Space Flight Center, Greenbelt, MD 20771 USA; Lasers & Electro-Optics Branch, NASA Goddard Space Flight Center, Greenbelt, MD 20771 USA; Lasers & Electro-Optics Branch, NASA Goddard Space Flight Center, Greenbelt, MD 20771 USA; Lasers & Electro-Optics Branch, NASA Goddard Space Flight Center, Greenbelt, MD 20771 USA; Lasers & Electro-Optics Branch, NASA Goddard Space Flight Center, Greenbelt, MD 20771 USA; Lasers & Electro-Optics Branch, NASA Goddard Space Flight Center, Greenbelt, MD 20771 USA; Lasers & Electro-Optics Branch, NASA Goddard Space Flight Center, Greenbelt, MD 20771 USA; Lasers & Electro-Optics Branch, NASA Goddard Space Flight Center, Greenbelt, MD 20771 USA; Lasers & Electro-Optics Branch, NASA Goddard Space Flight Center, Greenbelt, MD 20771 USA; Headquarters, Science Systems and Applications Inc., Lanham, MD 20706-6239 USA; Headquarters, Science Systems and Applications Inc., Lanham, MD 20706-6239 USA

**Keywords:** Fiber amplifier, fiber laser, lidar, lidar instrument, remote sensing, solid-state lasers, space instrument, space laser

## Abstract

At NASA Goddard Space Flight Center, we have been developing spaceborne lidar instruments for space sciences. We have successfully flown several missions in the past based on mature diode pumped solid-state laser transmitters. In recent years, we have been developing advanced laser technologies for applications such as laser spectroscopy, laser communications, and interferometry. In this article, we will discuss recent experimental progress on these systems and instrument prototypes for ongoing development.

## I. Introduction

IN THE past 20+ years, we have successfully developed and flown lidars [1]–[3] for mapping Mars [4]–[7], Earth [8], [9], Mercury [10], and the Moon [11] based on diode-pump solid state laser (DPSSL) technology [12]–[16]. More recently, two Earth orbiting laser-based missions were launched to continue Earth observing science applications based on DPSSL technology [17]–[21]. As laser and electro-optics technologies continue to expand and mature, more sophisticated instruments that once were thought to be too complicated for space are being considered and developed. Demand for wavelengths spanning from ultraviolet (UV) to mid-infrared (MIR) to meet a broad range of science goals as well as continual development of instruments for smaller satellite platforms are high and often cannot be easily met using traditional DPSSL. We are developing several new, space-based laser instruments that involve DPSSL and fiber-based laser technologies to satisfy a vast variety of remote sensing missions. These include lidars for remote sensing of carbon dioxide and methane on Earth for carbon cycle and global climate change; laser communications; gravitational wave detection based on laser ranging interferometers; a new generation of multiwavelength laser altimetry; and *in situ* laser instruments including potential use of ultra-short pulse generation for time-of-flight mass spectrometer to study the diversity and structure of nonvolatile organics in solid samples on missions to outer planetary satellites and small bodies.

We have successfully flown missions based on DPSSL for scientific measurements. These types of laser system designs have space heritage, can be easily scaled for specific use, and can be ruggedized for space deployment. At the same time, as we develop DPSSL for space, we are also investing in fiber laser and amplifier technologies. Fiber laser and amplifier systems have captured a large market share in recent years due to the ever increasing demands of materials processing applications, which include automobile, shipbuilding, pipeline laying, construction, electronics, and aerospace. Fiber lasers and amplifiers have the potential for superior beam quality (TEM_00_), high electrical-to-optical efficiency (>30% wall-plug), lower maintenance, higher reliability, smaller footprint, ruggedness, and easier transportability when compared to traditional DPSSL systems. Regardless of the approaches, critical attributes that are most relevant to NASA’s future missions include the following

1. Low susceptibility to optical misalignment and contamination.
2. Highly reliable active and passive components.
3. Availability of radiation-tolerant devices and components.
4. High wall-plug efficiency.
5. Small size, weight, and power (SWaP).

Although mJ level pulse energies have been reported in fiber lasers [22], in general, the pulsed fiber-laser/amplifier optical-peak-power is much lower than what is available from bulk solid-state lasers. Thus, new and optimized system architectures and measurement approaches are required to exploit and optimize the device capabilities. Rather than low pulse-repetition-frequency (PRF) (1–100 Hz) high-peak-power systems, we are investigating high-PRF modest peak-power instruments for new space instrumentation. Indeed, the Ice, Cloud, and Land Elevation Satellite 2 (ICESat-2) mission adapted the use of a micro-pulse lidar approach in using a high repetition rate, lower pulse energy laser to meet the science objectives [17]–[19]. For some of the applications, especially for satellites orbiting planets that have an atmosphere, backscattering from the atmosphere may restrict the PRF of the laser transmitter due to range ambiguity. A new measuring scheme is necessary to accommodate the use of transmitters that prefer high PRF operations.

SWaP requirements as well as hardware reliability are strong factors in determining the viability of a space-based instrument. Although each application has numerous factors that influence technology decisions, a high wall-plug efficiency system allows fewer components and provides margins for a given optical output power, allowing for higher derating and improved reliability. This is especially true for smaller satellites such as SmallSat or CubeSat [23] for future mission opportunities where resources are limited.

## II. Current and Upcoming Flight Missions

Recent launched missions described in first part of this section used time-of-flight measurement schemes for Earth science applications. The Global Ecosystems Dynamic Investigation mission uses low PRF, high laser pulse energy for the measurement while the ICESat-2 uses the micropulse lidar approach where the PRF of the laser is high and low pulse energies approach. This is the first micropulse lidar flown by NASA Goddard Space Flight Center (GSFC) for Earth science application.

### A. Global Ecosystems Dynamics Investigation (GEDI)

**Fig. 1.**
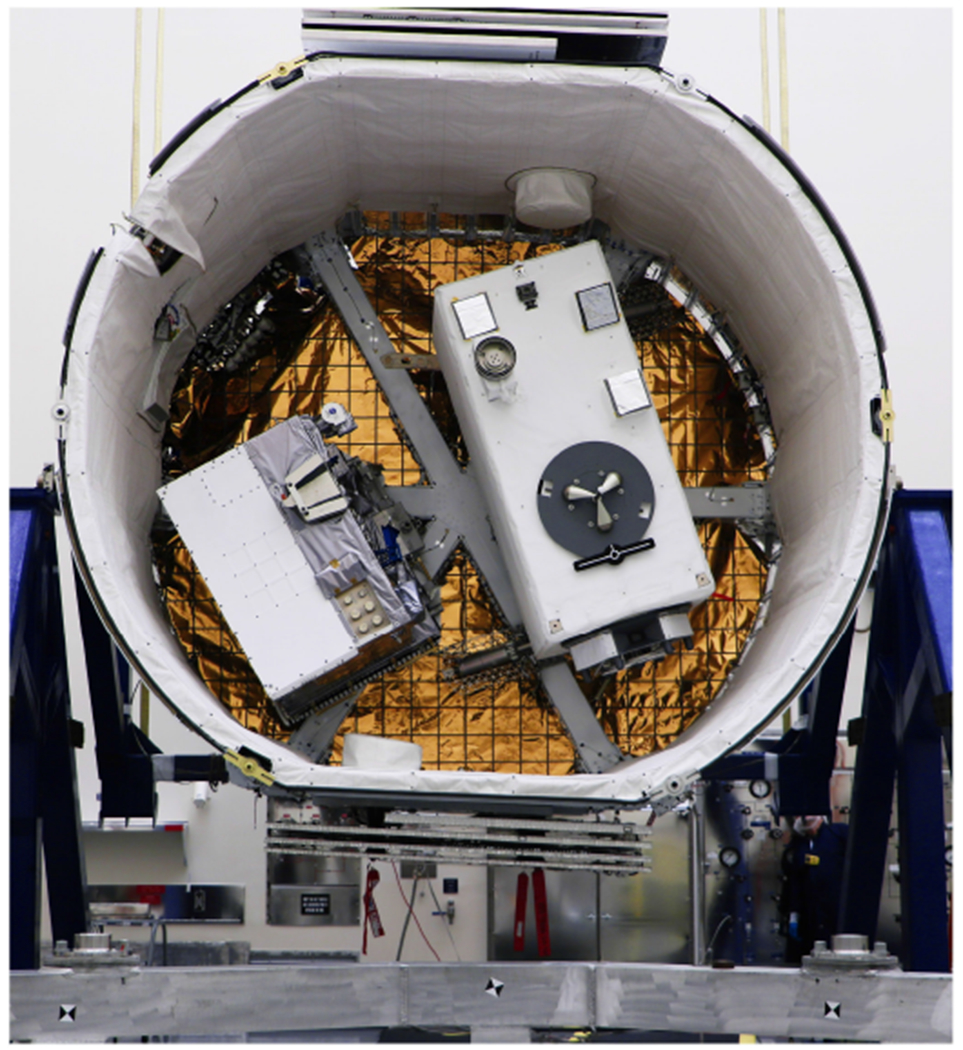
GEDI installed in JEM.

The GEDI lidar, as shown in Fig. 1, is an Earth Science remote sensing instrument aboard the International Space Station (ISS) and the Japanese Experiment Module. Its core mission is to measure the global carbon balance of Earth’s forests by using a set of three solid state laser transmitters in a multibeam waveform capture lidar technique. GEDI’s laser transmitters and precision optical system transmits over 3.4 million laser pulses to the Earth every hour, each pulse producing an individual three-dimensional (3-D) biomass column measurement. To enable a successful two-year mission, the lasers had to be reliable, highly repeatable in performance with each measurement power cycle, and designed with minimal part count for reduced manufacturing complexity and cost. These transmitters are in-house products; developed, constructed, qualified, and fully integrated into the GEDI instrument at NASA GSFC [21], [24]–[26].

GEDI has been operating successfully in orbit since January 2019. GEDI employs three Q-switched Nd:YAG laser transmitters, designed for maximum lifetime with highly derated diode array pump sources and carefully managed internal cavity fluences and temporal stability. They each operate independently at 242 Hz, producing 10 ns 1064 nm pulses between 10 and 11 mJ. In order to produce three sets of staggered footprints on the Earth’s surface, maximizing coverage and data set consistency, each laser is coupled to an external beam dithering unit (BDU) comprised of a KD*P Pockels cell pair, waveplate, and birefringent wedge. The BDUs produce a fixed angle-deflection at 121 Hz, or every “other” pulse. These footprints are producing precise measurements of the forest canopy height, canopy vertical structure, and surface elevation over a two-year mission baseline, recently extended to a third year and possibly more. At the two year mark, each laser has produced over 7.6 Billion measurements with almost no degradation, and adjustments needed to performance.

### B. Ice, Cloud, and Land Elevation Satellite-2 (ICESat2)

**Fig. 2.**
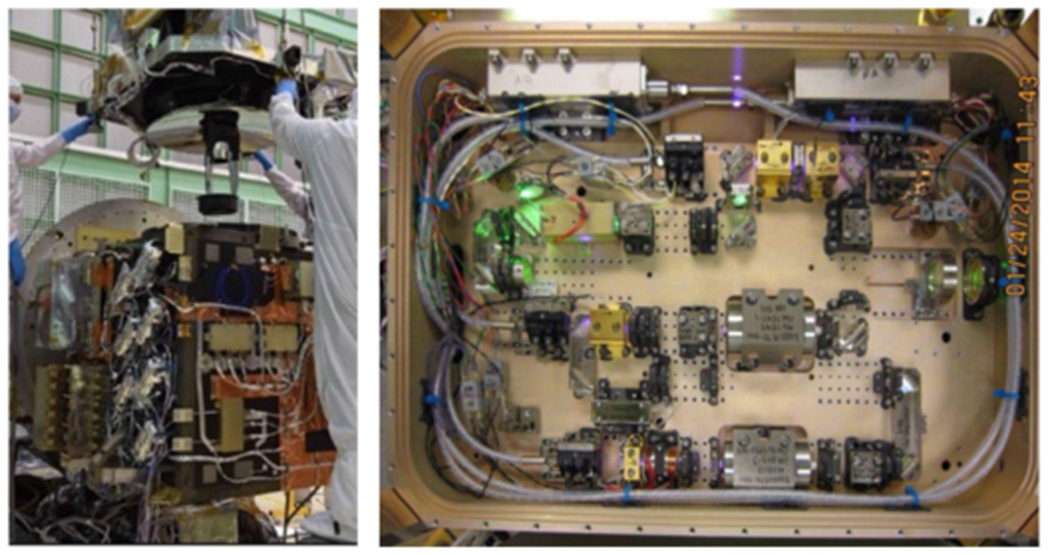
(Left) Two ATLAS lasers mounted on the ATLAS instrument during Integration and testing; (Right) Top view of the ATLAS laser optical module.

ICESat-2 is a follow-on to the ICESat mission [27], which was launched in January 2003 and concluded operations in February 2010. The ICESat-2 satellite mission was launched into the low Earth orbit in September 2018 from Vandenberg Air Force Base in California, USA. The sole instrument is the photon-counting lidar Advanced Topographic Laser Altimeter System (ATLAS) and has been in operation in space for over two years [28]. ICESat-2 is a satellite mission for measuring ice sheet elevation and sea ice thickness, as well as land topography, vegetation characteristics, and clouds on Earth (see Fig. 2). It provides high-quality topographic measurements that enable estimates of ice sheet volume change and from that estimation, the contribution of ice sheet melting to sea level rise. The high-accuracy altimetry also provides valuable information for making long-sought repeat estimates of sea ice freeboard and hence sea ice thickness change, which is used to estimate the flux of low-salinity ice out of the Arctic basin and into the marginal seas. The third objective of the mission is to produce globally distributed measurements of vegetation height to improve estimates of terrestrial above ground biomass [29].

The micropulse laser altimeter system for ATLAS represents a new space-based altimeter architecture. Similar altimetry systems utilizing high repetition rate, low energy pulses, multiple wavelengths, multiple beams, and single-photon ranging were successfully flown on airborne platforms recently [30]–[32]. The ICESat-2 observatory and ATLAS instrument use a photon-counting lidar and ancillary systems (primarily GPS and star cameras) to make three primary measurements—1) the time of flight of a photon from ATLAS to the earth, and back to ATLAS; 2) the pointing vector at the time a photon is transmitted by ATLAS; and 3) the position of ICESat-2 in space at the time a photon is recorded by ATLAS.

The key requirements for the ATLAS laser are summarized in Table I. All previous laser altimeters for space have been quasi-continuous wave laser diode array pumped solid-state oscillator/amplifier systems with low PRF and high energy with a single-beam footprint [except for the Lunar Orbiter Laser Altimeter (LOLA) [11] mission].

**Fig. 3.**
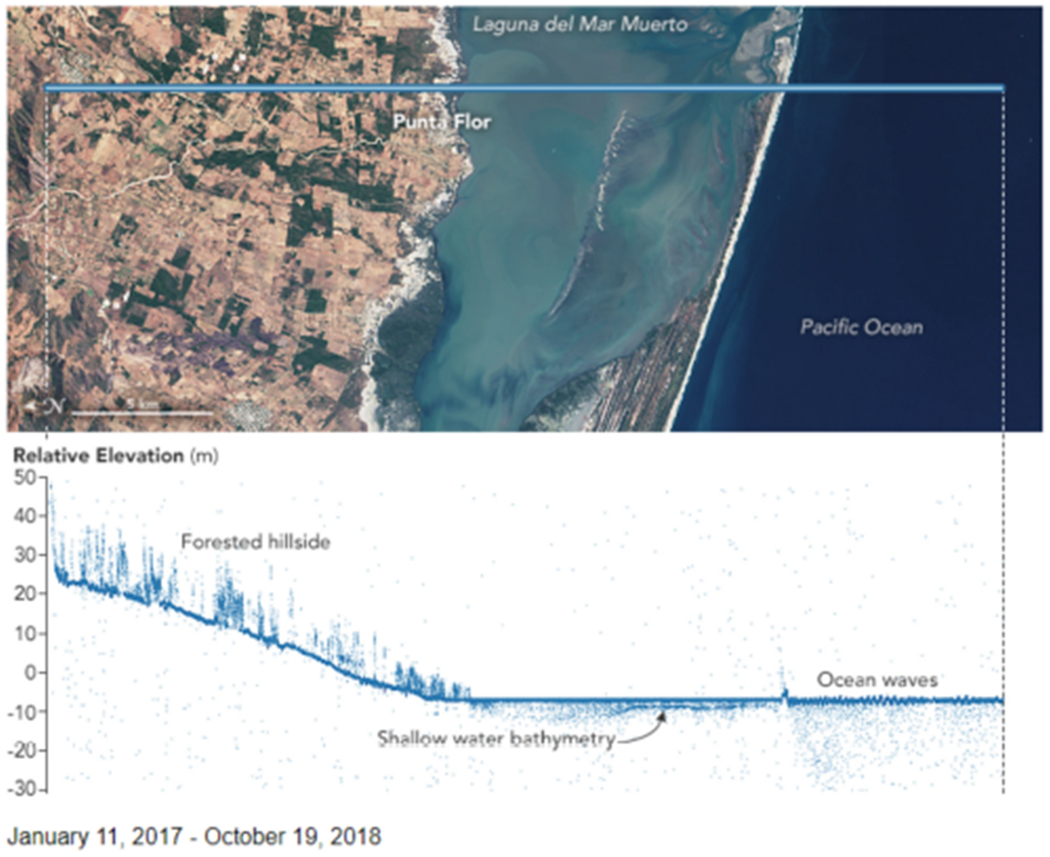
ICESat-2 sees the trees in Mexico.^1^

For ATLAS, the frequency-doubled Nd:YVO_4_ laser operates at 10 kHz PRF with a pulsewidth approximately 1.3 ns at full width half maximum and pulse energy commandable in 12 steps between 250 and 1400 *μ*J; it is currently operating with a pulse energy about 500 *μ*J. NASA’s ICESat-2 mission is meeting its measurement performance requirements [33], [34] (see Fig. 3 for sample science products) and is on track to continue meeting them for at least the required three years.

### C. New Frontiers—Dragonfly

The Dragonfly Mass Spectrometer (DraMS) is an airborne rotorcraft under development by the Applied Physics Lab to investigate multiple surface sights for Saturn’s moon of Titan and its landscape and atmosphere [35]. Aboard the radioisotope powered octo-copter is the DraMS, a NASA-GSFC developed instrument employing soil sampling and processing capabilities with pyrolysis and gas chromatography, as well as laser desorption mass spectrometry (LDMS). At the heart of the LDMS is a compact, solid state UV laser source, designed for stable burst mode operation, capable of delivering selectable pulse energies with high precision at 266 nm at < 2 ns pulsewidths. These pulses, between 10–200 *μ*J, will be used to excite the processed surface samples and produce molecular ions for injection into the mass spectrometer ion trap for prebiotic analysis. The UV laser is an optimized, end pumped Nd:YAG passive Q-switched cavity, with inherent single frequency oscillation, and external electro-optic polarization control of the fractional 1064 nm photons [36]. This capability enables the fractional pulse portions to be directed into the nonlinear optics for 266 nm production. Each burst, typically 1–50 pulses @ 100 Hz for ≤ 0.5 s, are preprogrammed and induces neither effect on pulse shape, beam quality, nor pointing for optimized and consistent ion clouds. Set to launch in 2026, this pressurized laser transmitter will need to survive a nine-year cruise and operate in the cold nitrogen rich atmosphere with surface temperatures around −200 °C.

### D. Laser Communication Relay Demonstration (LCRD)

**Fig. 4.**
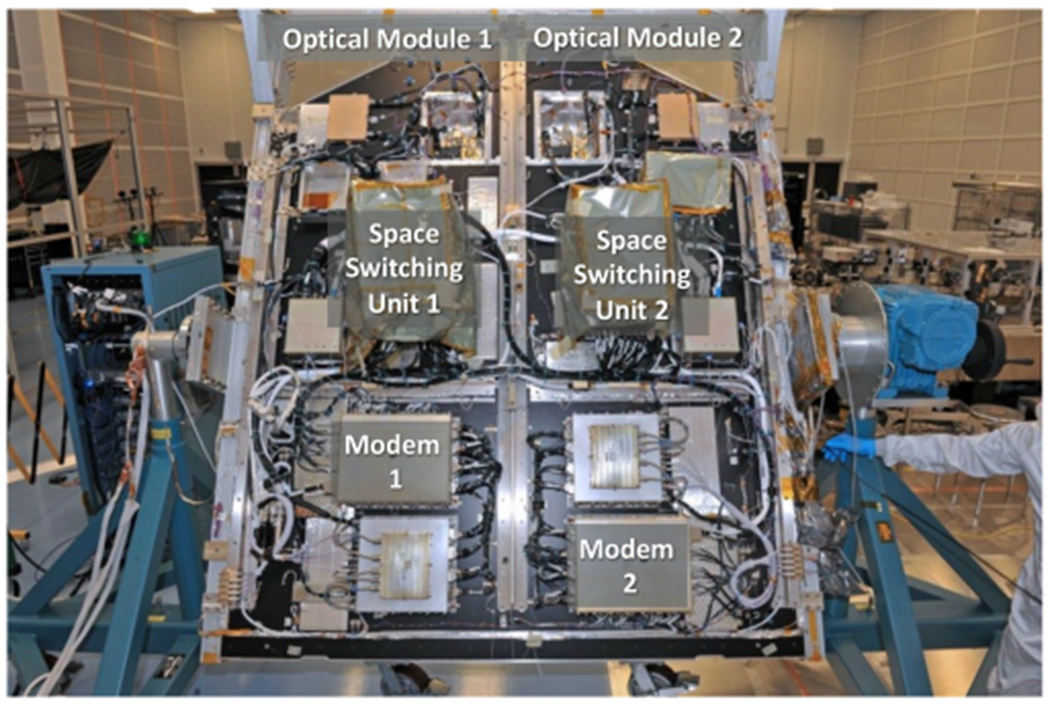
Integrated LCRD payload at NASA Goddard Space Flight Center.

We recently delivered the LCRD flight terminals (Fig. 4) to the project for launch in 2021. Each terminal uses a telecommunication laser diode master oscillator (MO) seeding an Er-doped fiber power amplifier (PA).

The LCRD terminals operate in the 1550 nm band and fly on a geosynchronous satellite. Each LCRD flight terminal consists of an optical module beam director, a controller electronics module, and a modem. Each modem supports both pulse position modulation and multirate software-defined differential phase shift keying formats with up to 2.88 Gbps data rate (1.244 Gbps user rate). The payload also has a 622 Mbps Ka-band RF downlink. There is a high speed switching unit to interconnect the two flight terminals. Each terminal communicates bidirectionally with one of the two optical ground stations located at California and Hawaii [37], [38]. The very-large-mode-area (VLMA) laser amplifier for the ground station leverages the development effort under the CO_2_ lidar laser transmitter described in the next section of this article.

## III. Laser Systems for Future Space Applications

### A. Trace Gas Sensing

We are maturing the technology of a laser transmitter designed for use in atmospheric carbon dioxide remote sensing. The ultimate goal is to make space-based satellite measurements with global coverage. In this program, we are working on a fiber-based master oscillator power amplifier (MOPA) laser transmitter architecture. The seed laser is a wavelength-locked, single frequency, externally modulated distributed Bragg reflector operating at 1572 nm followed by Er-doped amplifiers.

**Fig. 5.**
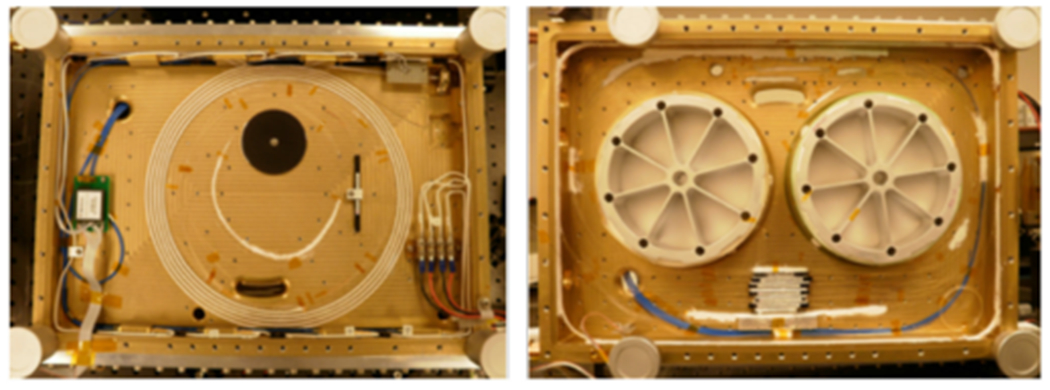
(Left) Top half of the two-sided enclosure with the PM-VLMA fiber. (Right) Bottom half of the enclosure with the 1480 nm Raman pump system. Module dimension is 44 cm × 32 cm × 9 cm.

The last amplifier stage is a polarization-maintaining, VLMA (PM-VLMA) fiber with ~1000 square microns effective area pumped by a Raman fiber laser as shown in Fig. 5. The optical output is single-frequency, one microsecond pulses with >450 *μ*J pulse energy, 7.5 kHz PRF, single spatial mode, and >20 dB polarization extinction ratio [39].

The global and regional quantification of methane fluxes and identification of its sources and sinks has been highlighted as one of the goals of the 2017 Earth Science Decadal Survey. Detecting methane from space with an active (laser) remote sensing instrument presents several unique technology and measurement challenges. The instrument must have a single frequency, narrow-linewidth light source, and photon-sensitive detector at the right spectral region to make continuous measurements from orbit, day, and night, in all seasons and at all latitudes. It must have a high signal to noise ratio and must be relatively immune to biases from aerosol/cloud scattering, spectroscopic and meteorological data uncertainties, and instrument systematic errors. The technology needed for a spaceborne mission is currently being developed by NASA and industry. At GSFC, we have developed an airborne instrument to measure methane. Our instrument is a nadir-viewing lidar that uses integrated path differential absorption (IPDA), to measure a methane vibration-rotational line near 1.65 *μ*m that is relatively free of interferences from other trace gases. We sample the absorption line using multiple wavelengths from a narrow linewidth laser source and a sensitive photodetector. The multiwavelength measurement approach minimizes biases in the CH_4_ retrievals [40].

**Fig. 6.**
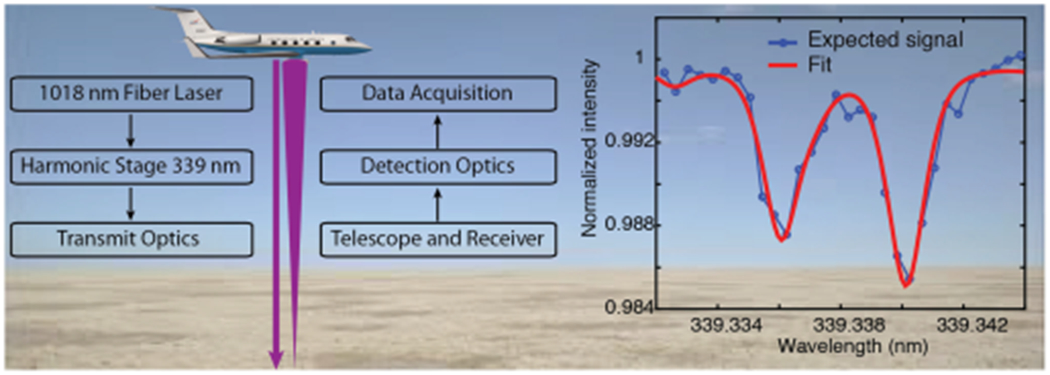
IPDA lidar uses a tunable laser to measure HCHO with absorption spectroscopy. Instrument includes a tunable laser, a reference cell for HCHO, and a transceiver samples the return signal from the ground.

Understanding the reactive photochemistry of Formaldehyde (HCHO) allows us to learn about the lifetime of greenhouse gases like methane, the production of ozone, and the growth of secondary organic aerosols which is critical to NASA’s Earth Science goals. As part of the instrument incubator program (IIP) funded by the Earth Science Technology Office (ESTO), we are developing a laser system that will make this measurement by employing a new method to detect formaldehyde remotely with IPDA lidar [41], [42]. This system will make use a tunable narrow-linewidth fiber-amplified laser to measure the absorbance of single rotational lines. The concept, as shown in Fig. 6, will measure the column of formaldehyde in the laser path using a simple Beer’s law analysis that is largely independent of the *a priori* assumptions needed in passive systems [43], [44]. We will also address the challenge of being able to detect the low abundances of HCHO in the UV where Rayleigh scattering is large.

A fiber optic-based architecture is well suited to meet these specifications as the technology offers high average power handling, flexibility in pulsewidth, PRF, etc., fusion-spliced construction for mechanical robustness, and near diffraction-limited beam quality.

**Fig. 7.**
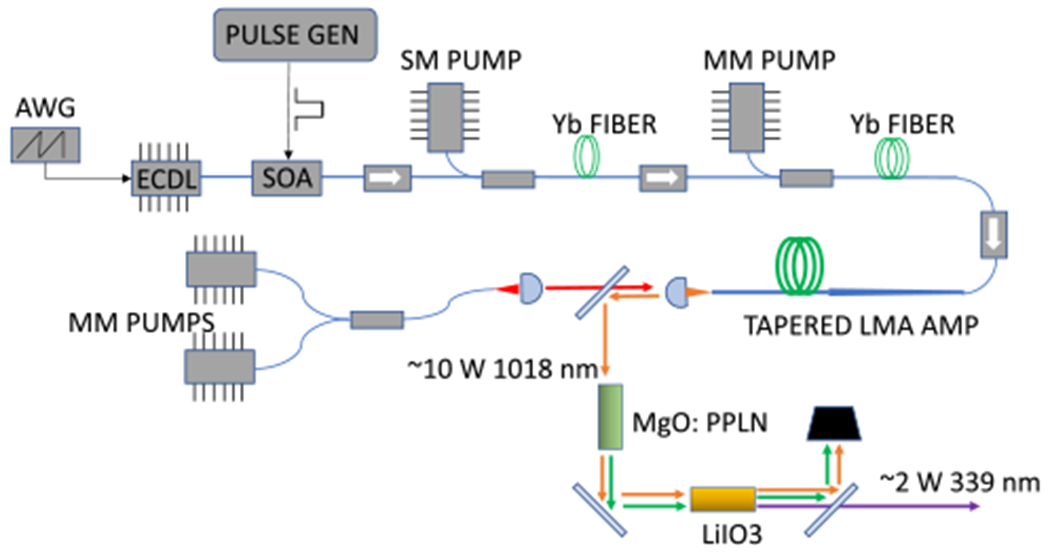
Block diagram of HCHO IPDA laser transmitter.

Furthermore, recent technology advances in large mode area (LMA) rare earth-doped fibers have made it possible to amplify spectrally narrow optical signals to high powers with excellent beam quality. Our laser system will leverage such advances made in ytterbium (~1 *μ*m) LMA and photonic crystal fibers, as well as decades of telecommunications investment in active and passive fiber components. We will use a two-stage power fiber amplifier to amplify the tunable, pulsed output from a 1018 nm external cavity diode laser, while a pair of nonlinear crystals will be used to first double the IR output to 509 nm and then mix the remaining fundamental with the 509 nm output to obtain 339 nm radiation. The block diagram of the laser transmitter is shown in Fig. 7.

### B. Astrophysics

We are developing an MOPA laser transmitter for the European Space Agency led Laser Interferometer Space Antenna (LISA) mission [45]. The LISA mission is a dedicated space-based gravitational wave detector, which aims to measure gravitational waves directly by using laser interferometry. As such, the laser requirements are particularly stringent in terms of laser frequency and intensity noises. Taking advantage of our space laser experience and the emerging telecom laser technology, we are developing a full laser system for the LISA mission. Our research effort has included both MO and PA developments, and their environmental testing and reliability for space flight. Our current baseline for the MO is a low-mass, compact micro nonplanar ring oscillator (*μ*-NPRO) laser. The amplifier uses a robust mechanical design based on optical fiber components. We have performed laser system noise tests by amplitude- and frequency-stabilizing the PA output. We are developing a technology readiness level (TRL) six laser system, which is an essential step toward qualifying lasers for space applications, by 2022 [46], [47].

**Fig. 8.**
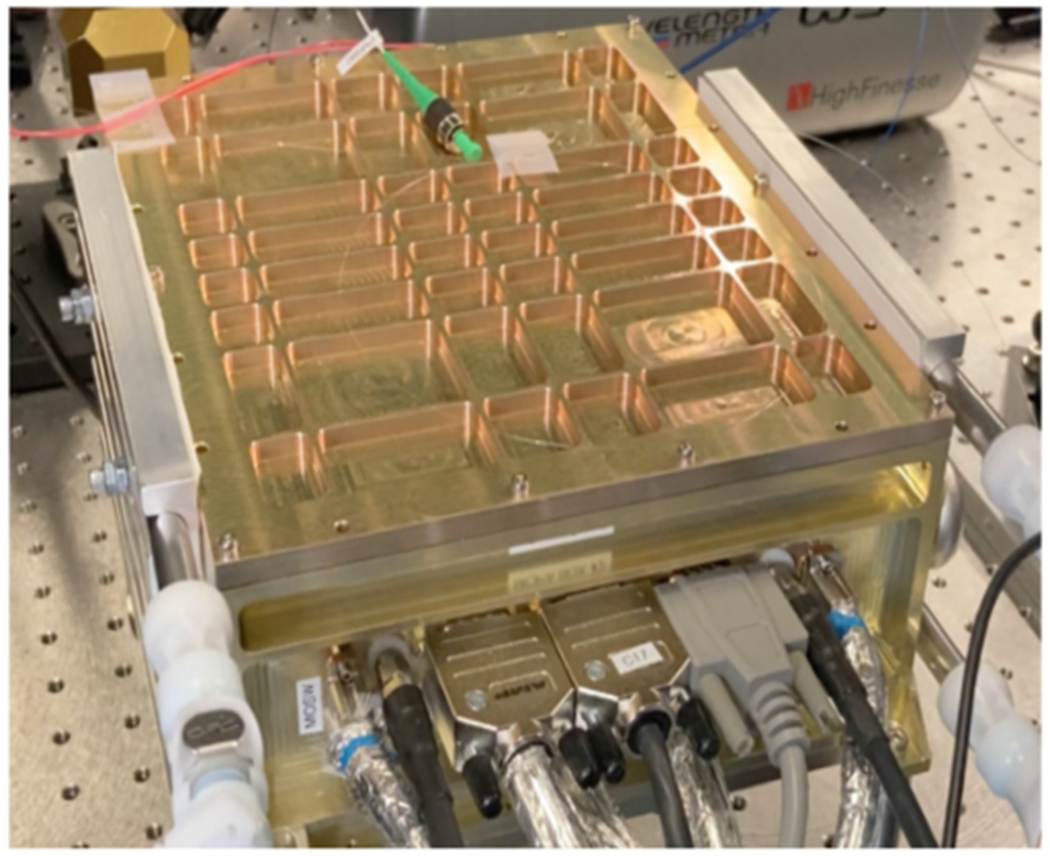
MOPA laser for the LISA mission.

The MOPA laser is packaged in a laser enclosure with dimensions of 240 mm × 195 mm × 98 mm and is shown in Fig. 8. The laser enclosure is two-sided with a center mounting surface. On one side of the enclosure, there are two *μ*-NPROs MO and two phase modulators (PM) provide full redundancy for the mission. These are connected to a 2×1 optical switch that provides selectivity of the MO and PM for the PA, which is mounted on the other side of the center mounting surface. Internal to the *μ*-NPRO, there are two 808 nm pump diodes, polarization combined to pump an NPRO crystal, both 808 nm pump diodes are nominally operating at >50% derating to meet the operational requirement. The output of the PA follows by an output high power optical isolator. The PA subassembly consists of a radiation hardened ytterbium polarization maintaining gain fiber pumped by a single 976 nm pump diode with two full redundant diodes via a tapered fiber bundle in the subassembly. Output of the MOPA is nominally 2 W average power to meet systems requirements. One of the most challenging requirements of the LISA laser is the long lifetime requirement, including on-ground testing, a 16-year lifetime is placed on the laser system. Full redundancy and significant derating on critical components, especially the 808 nm pump diodes for the *μ*NPROs and 976 nm pump diodes for the PA are used to meet the reliability requirement.

### C. Heliophysics

We proposed a space-based Na Doppler resonance fluorescence LIDAR for the measurement of temperature and vertical wind of the Earth Mesosphere Lower Thermosphere (MLT) 75–115 km region using atomic sodium as a tracer. The Atmospheric Coupling and Dynamics Across the Mesopause (ACaDAMe) mission concept uses a high-energy laser transmitter at 589 nm and highly sensitive photon counting detectors that permit range-resolved atmospheric-sodium-temperature profiles [48]. The atmospheric temperature is deduced from the linewidth of the resonant fluorescence from the atomic sodium vapor D_2_ line as measured by our tunable laser. Currently, we are developing a high-altitude Balloon-borne Sodium lidar to measure Tides in the Antarctic Region (B-SoLiTARe) for Earth mesosphere temperature measurements. The B-SoLiTARe instrument will focus on studying the largely unknown tidal structure in the upper polar mesosphere and would complement a future ACaDAMe mission by performing measurements at latitudes unreachable by the ISS. We are pursuing high power laser architectures that permit daytime sodium lidar observations with the help of a narrow bandpass etalon filter. There is no simple laser architecture to generate the 589 nm we need for sodium spectroscopy. Our two 589 nm wavelength laser architectures are 1) Raman laser for the B-SoLiTARe instrument; and 2) sum frequency generation (SFG) for the ACaDAMe mission.

We have demonstrated a narrow linewidth intracavity Raman laser operation at the sodium D_2_ line [49]. A Q-switched, diode pumped, c-cut Nd:YVO_4_ laser has been designed to emit a fundamental wavelength at 1066.6 nm. This fundamental wavelength is used as the pump in an intracavity Raman conversion in a Gd_0.2_ Y_0.8_ VO_4_ composite material. By fine tuning the mixture ratio, x, in our custom mixed Raman crystal Gd_x_Y_(1-_*x*_)_VO_4_ with *x* = 0.2, we have successfully generated the desired Raman shifted wavelength to Na resonance lines. We are continuing our efforts of rapid frequency tuning and power scaling to meet the laser requirement for B-SoLiTARe instrument shown in Table II.

The Raman laser is injection seeded with both 1066.6 and 1178 nm for single frequency output [49]. To rapidly step through the three desired laser wavelengths across the Na D_2_ line near 589 nm, we injection seed the Raman laser with an electronically tunable seed laser near 1178 nm. The Raman laser will tune correspondingly. The wavelength stability of the 589 nm laser pulses is achieved by locking the wavelength of the injection seeder to a Na vapor cell at 589 nm using a saturation-spectroscopy technique. Its frequency drift can be suppressed to sub MHz at 1 s averaging time.

**Fig. 9.**
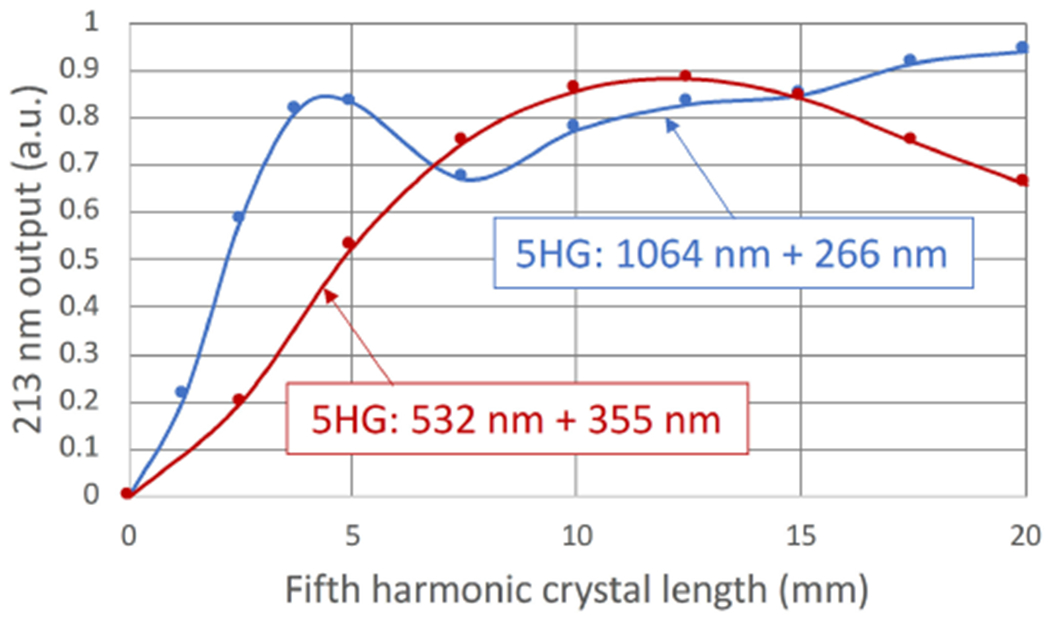
5HG of 213 nm as function of crystal length.

In a parallel effort, we have demonstrated power scaling of the Raman laser. An 880 nm pumping scheme is selected to reduce the thermal load to the Nd:YVO_4_ crystal. We have built a Z-shaped shared cavity Raman laser similar as Fig. 9(a) in our previous report [49], with the modification of only one side 880 nm pump and no intracavity doubling crystal. We obtained 2.2 W output at 1178 nm and expected to have 1.5 W at 589 nm with external frequency doubling. For a single frequency and high output power operation of a c-cut Nd:YVO_4_ laser in linear cavity configuration, thermal depolarization and spatial hole burning need to be carefully addressed. The twisted mode cavity configure can effectively eliminate the spatial hole burning and reduce the thermal depolarization for moderate output power operation of end pumped c-cut Nd:YVO_4_ laser. As a better alternative, a ring laser cavity uses an a-cut Nd:YVO_4_ crystals operating in s-polarization for 1178 nm Raman laser has been proposed [49], [50]. The strong natural birefringence of the a-cut Nd:YVO_4_ crystal dominates the thermal induced birefringence and results in linear polarized lasing. A unidirectional ring laser eliminates the spatial hole burning. In the case of an a-cut Nd:YVO_4_ crystals operating in *σ*-polarization, it is necessary to suppress the *π*-polarization oscillation because the p-polarization cross section is four times higher than *σ*-polarization. A pair of Brewster cut Gd_0.2_ Y_0.8_VO_4_ mixed Raman crystals is inserted in the ring resonator at the smallest beam waist for highest Raman gain. The a-cut Nd:YVO_4_ gain crystal is placed where the mode size is large. In this design, the main Raman conversion will take place in the mixed Raman crystal at the desired Raman shifted wavelength. The exact wavelength and narrow linewidth will be controlled by the injection seedings both at 1066.6 nm fundamental and 1178 nm Raman radiation. The 1066.6 nm seed is tuned to lock on the laser cavity and the cavity is then tuned to locked on the frequency stabilized 1178 nm seed laser. The ring shared cavity Raman laser is currently under development.

## IV. Altimetry for Earth and Planetary Applications

We are developing a next generation space lidar system with low SWaP. The lidar transmitter is a single fiber laser with high PRF, high peak, and average optical power fiber laser. The time interleaved fast wavelength tuning technology is deployed in this novel design, which enables dynamic multiple ground track scanning for wide swath coverage of target. A high efficiency fiber optical amplifier with high peak and average power is a key component in the lidar transmitter for this development [51].

### A. *Mass and Laser Spectroscopy for Planetary* In-Situ *Lidar*

Planetary and small body lander missions continue to seek instrumentation that comprehensively characterize the composition of the planetary surface and/or near-subsurface materials. Laser mass spectrometry (LMS), advanced at GSFC over the years, is typically utilized to identify and characterize trace amounts of astrobiologically relevant organic content in the acquired samples, however its ability to provide geological context by identifying the composition of the inorganic fraction is generally limited.

Mass spectrometers represent progressive analytical platforms for future *in-situ* lander missions to explore the surface chemistry of planetary bodies. Europa (as an astrobiology objective) and the Moon (an extension of the terrestrial system) are two primary targets for future NASA missions to search for extraterrestrial life and potentially habitable environments beyond Earth, further our understanding of the timing and formation of the Solar System, and identify potentially viable economic resources such as water and/or valuable metal assets. The CORALS (Characterization of Ocean Residues and Life Signatures) [52], [53] and CRATER (Characterization of Regolith and Trace Economic Resources) instruments are laser-based Orbitrap™ mass spectrometers currently under development for prospective lander missions to Europa and the Moon, respectively. We are advancing two compact, robust, and high TRL UV solid state lasers that serve as the sampling and ionization sources of these two investigations. The UV sources are based on converting the fundamental output from previously flown laser transmitters for LOLA [16] and mercury laser altimeter (MLA) [15] by fourth (FHG) and fifth harmonic generation (5HG) for CORALS and CRATER, respectively [54].

iSEE (*in-situ* Spectroscopic Europa Explorer) is a next-generation ultra-compact Raman system with superior performance that meets the top-level scientific requirements of multiple planetary missions to the inner and outer Solar System that was previously funded under the NASA STTR program and is currently funded by the NASA Maturation of Instruments for Solar System Exploration (MatISSE) program. iSEE integrates, for the first time, a digital micromirror device/photomultiplier assembly and a microchip diode laser into a miniature Raman spectrometer that enables unprecedented measurements: *in-situ* chemical identification and quantitation of complex organic compounds, including prebiotic compounds (e.g., amino acids); biomolecules (organic biomarkers including proteins, lipids, and nucleic acid polymers); minerals/salts; and volatiles. iSEE also provides sample context, including ice composition, crystallinity, and ice phase distribution [55]. We are currently developing a laser transmitter for the iSEE Raman spectrometer that is a frequency doubled Yb:YAG microchip laser that was previously developed under an ESTO funded IIP [56].

RAMS (Raman Mass Spectrometer) is a hybrid instrument that incorporates LDMS and micro Raman spectroscopy imaging into a compact instrument package. The instrument benefits from shared resources to reduce the SWaP while simultaneously enabling comprehensive sample analysis capabilities transcending those of either technique alone. The RAMS hybrid instrument prototype will be capable of acquiring colocalized organic molecular and mineralogical composition maps with ~10 *μ*m spatial resolution, thus revealing the spatial associations between organic and mineral phases at the necessary level of detail to inform on the provenance of 1) prebiotic molecules on comets and asteroids as well as 2) potential molecular biosignatures on Ocean Worlds. We are developing a dual wavelength laser transmitter for the RAMS instrument that simultaneously generates both a visible (515 nm) and deep UV (DUV) (257.5 nm) output. The RAMS laser is a frequency doubled and quadrupled diode pumped Yb:YAG microchip laser [55] that generates 515 and 257.5 nm simultaneously. The laser transmitter requirements for CRATER, CORALS, iSEE, and RAMS are summarized in Table III.

The CRATER laser is a 1064 nm Nd:YAG MOPA based on the previously flown MLA [15] with a 5HG to produce >1 mJ pulse energy at 213 nm with a 5 ns pulsewidth. The fundamental laser operates at 1–10 Hz PRF and 20 mJ pulse energy. To generate the fifth harmonic, there are three frequency conversion stages. First, the 1064 nm is frequency doubled by second harmonic generation (SHG) using a Type-ILBO (lithium triborate) crystal to generate 532 nm. Then, SFG is used to combine the 532 nm beam with the residual 1064 nm beam in a Type-II LBO crystal for third harmonic generation of 355 nm. Finally, the 355 nm beam is mixed with the residual 532 nm beam in a Type-I BBO (beta barium borate) crystal for 5HG to 213 nm. A Pockels cell is used to rotate the polarization of the 532 nm beam prior to the 5HG BBO crystal to change the phase matching condition and vary the conversion efficiency to 213 nm. Additionally, the PA pump diode current of the MLA laser can be adjusted to provide coarse energy attenuation. Dichroic filters and dispersive prisms are used to separate the 213 nm output from the residual 1064, 532, and 355 nm light. A lens focuses the beam to the sample location in the CRATER instrument and a micro-electro-mechanical systems (MEMS) mirror is used to raster scan the beam over a 500 *μ*m × 500 *μ*m area to provide 2-D chemical mapping. The CRATER laser enclosure will be pressurized with >1 atm of clean dry air to reduce the risk of laser induced contamination damage.

There are two ways to generate 213 nm via 5HG. One approach is two consecutive frequency doubling stages for FHG to 266 nm followed by SFG between residual 1064 and 266 nm. The other, which is also our choice, is to frequency double 1064 nm to generate 532 nm and two SFG stages afterwards to generate 355 nm and then 213 nm from residual 532 and 355 nm. While the first approach may be less complicated, the presence of two DUV wavelengths (~200 to 280 nm) in the system may increase the chance of UV induced damage.

To evaluate the frequency conversion approaches and estimate the optimum crystal types and lengths of each stage, SNLO program (AS-Photonics, Inc) was employed for nonlinear optical modeling. The simulation was performed on each frequency conversion stage with varying crystal types and lengths, input energies and energy ratio (for SFG) in consideration with transverse beam offset ratios and walk-off angle. The simulation example shown in Fig. 9 is the trend of 213 nm output energy from 1064 nm+266 nm and 532 nm+355 nm approaches as the length of the 5HG crystal varies. In this simulation, the fundamental 1064 nm energy was 20 mJ and 1/e^2^ beam diameter was 1.7 mm, crystal lengths for the first and second conversion stages for both approaches were set at the same length and the resultant output and residual energies were carried over to the next conversion stage. The simulation results indicated that the proposed energy goal could be achieved from both approaches and the 532 nm+355 nm conversion was selected for CRATER laser to lower the possibility of UV induced optical damage.

The CORALS laser is a 1064 nm Nd:YAG MO based on the previously flown Geoscience Laser Altimeter System [14], MLA [15], and LOLA [16] oscillators with FHG to produce 450 *μ*J pulse energy at 266 nm with a 5 ns pulsewidth. The fundamental 1064 nm laser operates at a PRF of 1–10 Hz and 2.8 mJ pulse energy. The 1064 nm beam is frequency doubled to 532 nm using a Type-I LBO crystal and quadrupled to 266 nm using a Type I BBO crystal. A Pockels cell is used to rotate the polarization of the 532 nm light prior to the fourth harmonic BBO crystal to change the phase matching condition and vary the conversion efficiency to provide variable attenuation.

**Fig. 10.**
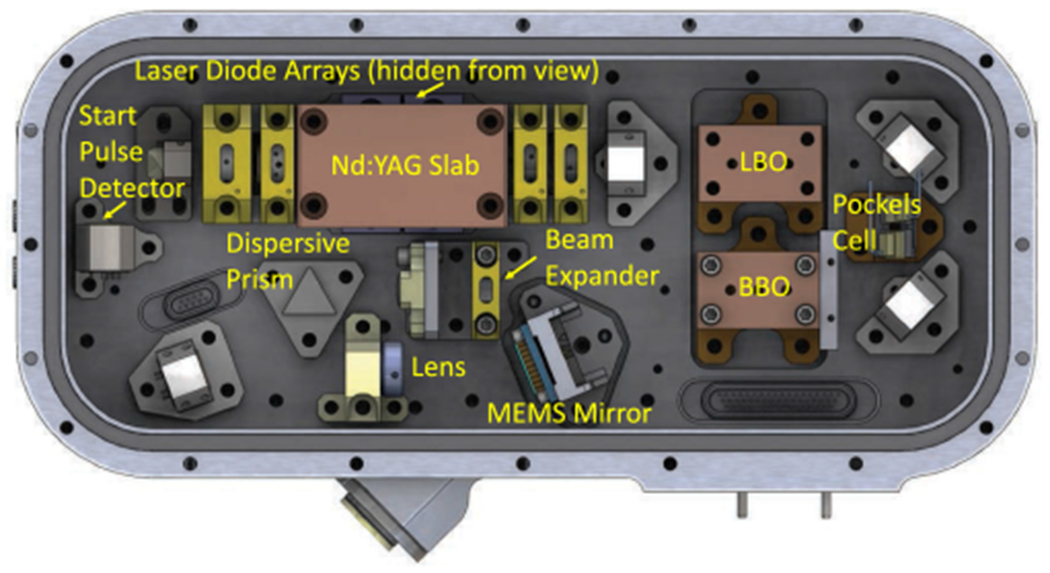
CORALS laser design.

Similar to the CRATER laser design the 266 nm light is separated from the residual 1064 and 532 nm light using a dispersive prism. A lens focuses the beam to a 50 *μ*m spot size at the sample location in the CORALS instrument and an MEMS mirror provides raster scanning of the beam over a 500 *μ*m X 500 *μ*m area. The CORALS laser enclosure is pressurized with >1 atm of clean dry air to reduce the risk of laser induced contamination damage. The CORALS laser is compact with dimensions 7.28 in × 3.33 in × 1.48 in and mass of < 1 kg. The CORALS laser model is shown in Fig. 10.

**Fig. 11.**
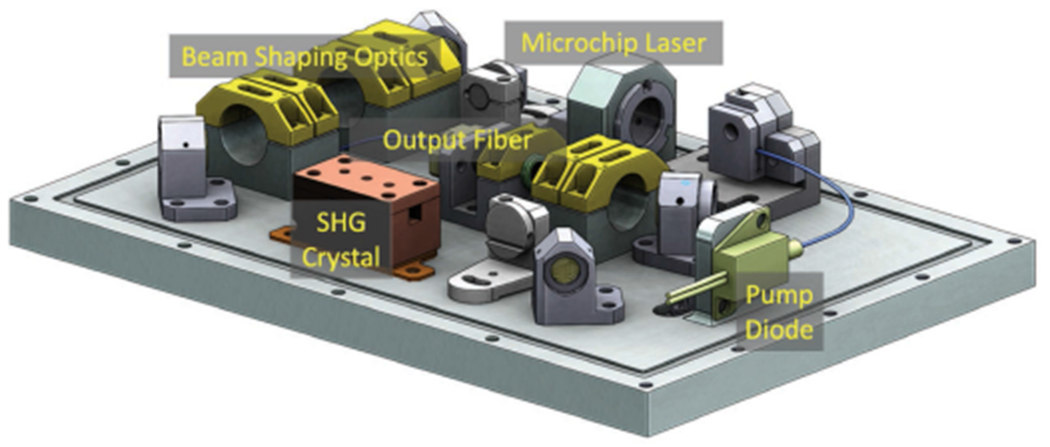
iSEE laser design.

The iSEE laser is a diode pumped 1030 nm Yb:YAG microchip laser with an SHG to achieve an output wavelength of 515 nm and fiber coupled to a 105 *μ*m core multimode fiber for delivery to the Raman spectrometer probe. The fundamental 1030 nm laser operates at a PRF of 1–10 kHz with pulse energy of 95 *μ*J and ~800 ps pulsewidth. A Type-II KTP crystal is used for SHG from 1030 to 515 nm. The residual 1030 nm is separated from the 515 nm using a pair of dichroic filters and a focusing lens is used to couple the 515 nm beam to the optical fiber. The output pulse energy from the fiber is 20 *μ*J at 515 nm. The Yb:YAG microchip laser was developed previously under a NASA ESTO IIP [56]. The iSEE laser is compact with dimensions of 5.7 in × 4.23 in × 2.28 in and <1 kg and pressurized with > 1 atm of clean dry air. The iSEE laser design is shown in Fig. 11.

**Fig. 12.**
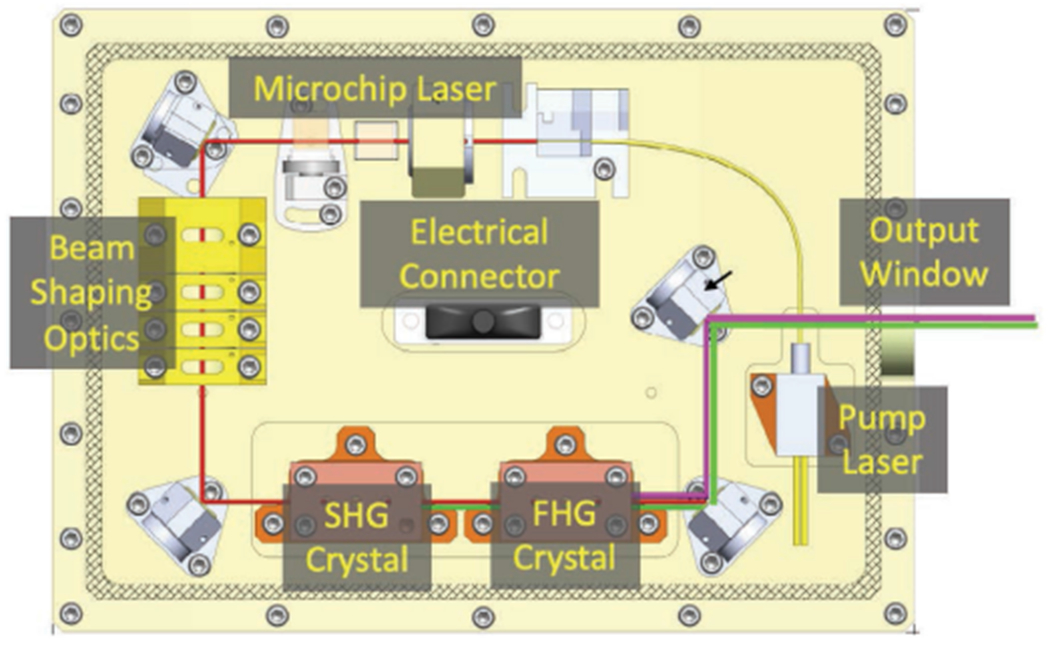
Preliminary RAMS laser design.

The RAMS laser is a diode pumped 1030 nm Yb:YAG microchip laser [56] similar to the iSEE laser with a SHG and FHG to achieve a visible and DUV output at 515 and 257.5 nm, respectively, the fundamental 1030 nm laser operates at a PRF of 1–10 kHz with pulse energy of 95 *μ*J and ~800 ps pulsewidth. A Type-II KTP crystal is used for SHG from 1030 to 515 nm, and a Type-I BBO crystal is used for (FHG) generation from 515 to 257.5 nm. Both the visible and DUV beams are coaligned and focused to the sample location in the RAMS instrument using an achromatic lens. An MEMS mirror is used to scan the laser beam in two dimensions over the sample. The preliminary RAMS laser prototype design is shown in Fig. 12.

In parallel, we are also investigating next generation laser architectures based on femtosecond (fs) laser to advance the capabilities of laser mass spectrometers, particularly for enhanced specificity to mineralogical rock composition and explore the potential of LMS for age dating rock samples on future planetary science missions. fs lasers are rapidly becoming an alternative to nanosecond or picosecond lasers for applications ranging from laser machining and surgeries to communications and material characterization. They owe their rise to the limited thermal and mechanical energy deposition into the probed material, which results in more precise material removal and reduced radiation damage. These characteristics make fs lasers particularly suitable for laser desorption/ionization time-of-flight mass spectrometry (LDMS) [57] and laser ablation MS [58], since reduced heating leads to reduced elemental fractionation and thus improved compositional accuracy, potentially enabling more accurate age dating of samples than available to date. We are particularly interested in fs laser technologies that would meet the systems requirements and have a path toward space deployment, especially one that offers a robust design with reduced SWaP. We began developing a fiber fs laser prototype that is based on the Mamyshev oscillator design [59]. This approach has the advantage of improved efficiency and a simplified architecture for space flight packaging and design compared to a mode-locked laser. For many LDMS applications, the required PRF is relatively low on the order of 1–10 Hz. Mode-locked lasers generate very high PRFs of ~100 MHZ. In the mode-locked laser case, a pulse picker can be used to produce the required PRF, but most of the laser energy is not used resulting in very low efficiency. In the Mamyshev oscillator design, we can set the required PRF with a gain-switched diode to provide a significant improvement in overall laser efficiency.

One of the more challenging requirements for planetary instrumentation is the dry heat microbial reduction (DHMR) protocol implemented to reduce the microbial bioburden on space-flight hardware prior to launch to meet flight project planetary protection requirements [60]. The sterilization process was achieved by subjecting hardware to elevated temperatures for more than 100 h. For our technology development programs, we subject our lasers to the DHMR process with a baked-out temperature of 115°C for 180 h [61] to access the laser robustness in meeting this protocol. Other temperature/time/pressure combinations are utilized based on mission requirements.

## V. Satellite Servicing

NASA GSFC is supporting the development of an advanced 3-D imaging lidar system now baselined for NASA’s Restore-L [62] project that will demonstrate an autonomous satellite-servicing capability. The 3-D imaging lidar, called the Kodiak system—formerly known as the Goddard Reconfigurable Solid-state Scanning Lidar, will provide real-time images and distance-ranging information during autonomous docking with satellite being serviced. This project will demonstrate how a specially equipped robotic servicer spacecraft can extend a satellite’s lifespan [63]. The laser requirements for the Kodiak system are summarized in Table IV.

The Safe and Precise Landing Integrated Capabilities Evolution (SPLICE) is part of the Game-Changing Development program at NASA that seeks to develop a new suite of guidance, navigation, and control technologies to enhance the landing capabilities of lunar descent vehicles. Future missions to the moon and other planets will rely on SPLICE technologies for the detection and avoidance of landing zone hazards such as boulders and craters. One such technology is the hazard detection lidar (HDL), a hybrid scanning-imaging lidar that performs rapid 3-D landing site imaging with real-time digital elevation map (DEM) generation for the identification of safe landing sites as well as hazard avoidance. HDL will generate high-density (~8 MPixel) DEMs of 100 m circular landing regions with cm-level vertical precision in 2 s or less.

**Fig. 13.**
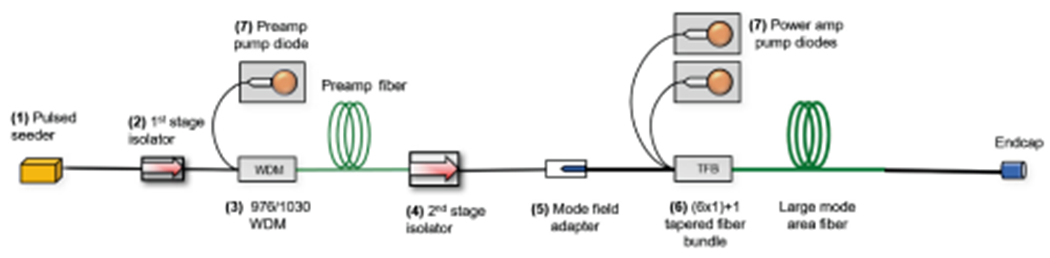
Block diagram of two-stage LMA fiber transmitter for HDL.

The instrument requires the laser transmitter to operate in a number of different modes: an altimeter mode for ranging at altitudes > 1 km (10 and 20 kHz) and a DEM acquisition/scanning mode (60, 120, and 240 kHz); all operating modes require 30 mJ/pulse energy and 4 ns pulsewidth to meet HDL requirements. A two-stage seeded YbLMA fiber amplifier architecture, shown in Fig. 13, has the flexibility needed to meet this wide range of operational modes with high efficiency and low SWaP requirements. We have developed a brassboard-level version of the laser that meets all the HDL requirements, and will deliver an environmentally-tested flight unit by the end of 2021.

## VI. Conclusion

NASA GSFC has been successful in deploying multiple Earth and planetary instruments to meet a wide range of science goals in the past decades. In the near future, diode pumped neodymium based near infrared lasers in the 1 *μ*m regime continued to dominate the space flight laser applications based on maturity, heritage, and more importantly, the availability of single photon sensitive detectors or detector arrays with high bandwidth that are necessary to meet system performances. A promising detector technology advancing by NASA GSFC with similar sensitivity will open up the wavelength choices for future space lidar applications [64]. Future demands on laser transmitters at specific wavelengths and spectral ranges are increasing as can be seen in examples in this article. For example, MIR wavelengths are receiving more attention for planetary applications as interests continue to grow in this area. Generation of these MIR wavelengths are based on parametric generation or amplification pumped with our heritage NIR lasers. Shorter wavelengths by harmonic generation based on our heritage lasers into the harmonics and especially UV also continues to grow as well. However, approaches are being sought to improve the wall plug efficiencies (WPE) of these systems. Typical WPE of existing laser systems is on order of a few percent, which in turns placed demands on the instrument resources such as solar panels. It is a fine balance between operating the laser efficiently and also provides enough margin or derating to meet mission lifetime. Reliability of laser-based instruments continues to be the ultimate concerns for spaceborne lidar systems. Derating and operation margins are two prime factors in designing instruments to meet mission goals for all agencies and research groups. We continue to leverage other industries and commercial investments for more reliable products to meet future science goals and objectives with the goals of lowering SWaP and increasing reliability. Main concerns for high-peak-power transmitters, in particular those that require narrow bandwidth and in some cases single-frequency operation, include laser damage of optics that could lead to irreversible damages and significantly shortened the life expectancy of these lasers. Contamination-induced laser damages, especially associated with UV lasers continued to limit the deployment of these lasers in space. Improved packaging approaches are needed to lower this risk for future missions. Fiber-based laser technologies are becoming more mature and appear to have a strong future for numerous NASA space-based instrument applications. In spite of the numerous advantages of fiber-based laser transmitters, there are still issues to be resolved for NASA applications. Other technologies that have seen tremendous growth in consumer market place such as small SWaP and low cost lidars for autonomous vehicles and hazard avoidance are examples that could help and accelerate growth in incorporating lidars in small-satellite or Cubesat platforms.

## Figures and Tables

**TABLE I:** Key Requirements for the ATLAS Laser Transmitter

Laser Transmitter Parameters	Values
Energy per pulse	250-900 μJ
Laser repetition rate	10 kHz
Pulse width	1.5 ns
Center wavelength	532.27nm
Linewidth (FWHM)	<30pm
Beam divergence	<130 μrad
Nominal operational run time	3 years and 2 months

**TABLE II:** Laser Requirements for B-SoLiTARe

Laser Transmitter Parameters	Value
Wavelengths	589.15900 nm 589.15846 nm 589.15790 nm
Average laser power	1 W
Laser pulse rate	10 kHz
Laser divergence angle	75 μrad

**TABLE III:** Laser Requirements for iSEE, RAMS, CORALS, and CRATER Programs

Programs	iSEE	RAMS		CORALS	CRATER
**Program**	MatISSE	PICASSO		ICEE 2	DALI
**Spectral Range**	**VIS**	**DUV**	**VIS**	**DUV**	**DUV**
**Period of Performance**	3 Years	3 Years		2 Years	3 Years
**Targeted Mission**	TBD - Planetary Astrobiology			Europa	Moon
**Mission Lifetime**	TBD	TBD		10 MShots	1 MShots
**Output Wavelength**	515 nm	257.5 nm	515 nm	266 nm	213 nm
**Pulse Energy**	20 μJ	10-20 μJ	20-40 μJ	>450 μJ	>1 mJ
**Rep Rate**	1-10 kHz	1-10 kHz (Selectable)		1-10 Hz	1-10 Hz
**Frequency Conversion**	SHG@ 1030 nm	FHG@ 1030 nm	SHG@ 1030 nm	FHG@ 1064 nm	5HG@ 1064 nm
**DHMR**	Yes	TBD		Yes	Yes
**Radiation**	TBD	TBD		300 krad	20 krad
**Heritage**	ESTO IIP [56]			GLAS [14], MLA [15], LOLA[16]	

**TABLE IV:** Kodiak System Laser Requirements

Requirements	Values
Operational Time (testing + on-orbit)	~ 1000 Hours
Center Wavelength	1553.xx nm ± 1.0 nm
Spectral Width	±0.5 nm
Wavelength Drift over Temperature	±3.0 nm and <1.0 nm per 10°C
Minimum Repetition Rate	100 kHz
Maximum Repetition Rate	200 kHz
Pulse Width	2.5 ns (TBR) ± 0.5ns
Peak Pulse Energy @ 100 kHz	3μJ ± 10%
Peak Pulse Energy @ 200 kHz	3μJ ± 10%
Dynamic Range	10 dB

## Footnotes

1 [Online]. Available: https://earthobservatory.nasa.gov/images/144450/icesat-2-sees-the-trees-in-mexico
